# Supplementary material for: The causal relationship between the human gut microbiota and pyogenic arthritis: a Mendelian randomization study
Source: Front Cell Infect Microbiol. 2024 Nov 26;14:1452480. doi: 10.3389/fcimb.2024.1452480 (PMC11629706; doi:10.3389/fcimb.2024.1452480)
Supplement: Supplementary file 1 [file DataSheet1.zip › mendelian test/ebi-a-GCST90027502.csv_leave_one_out.pdf]

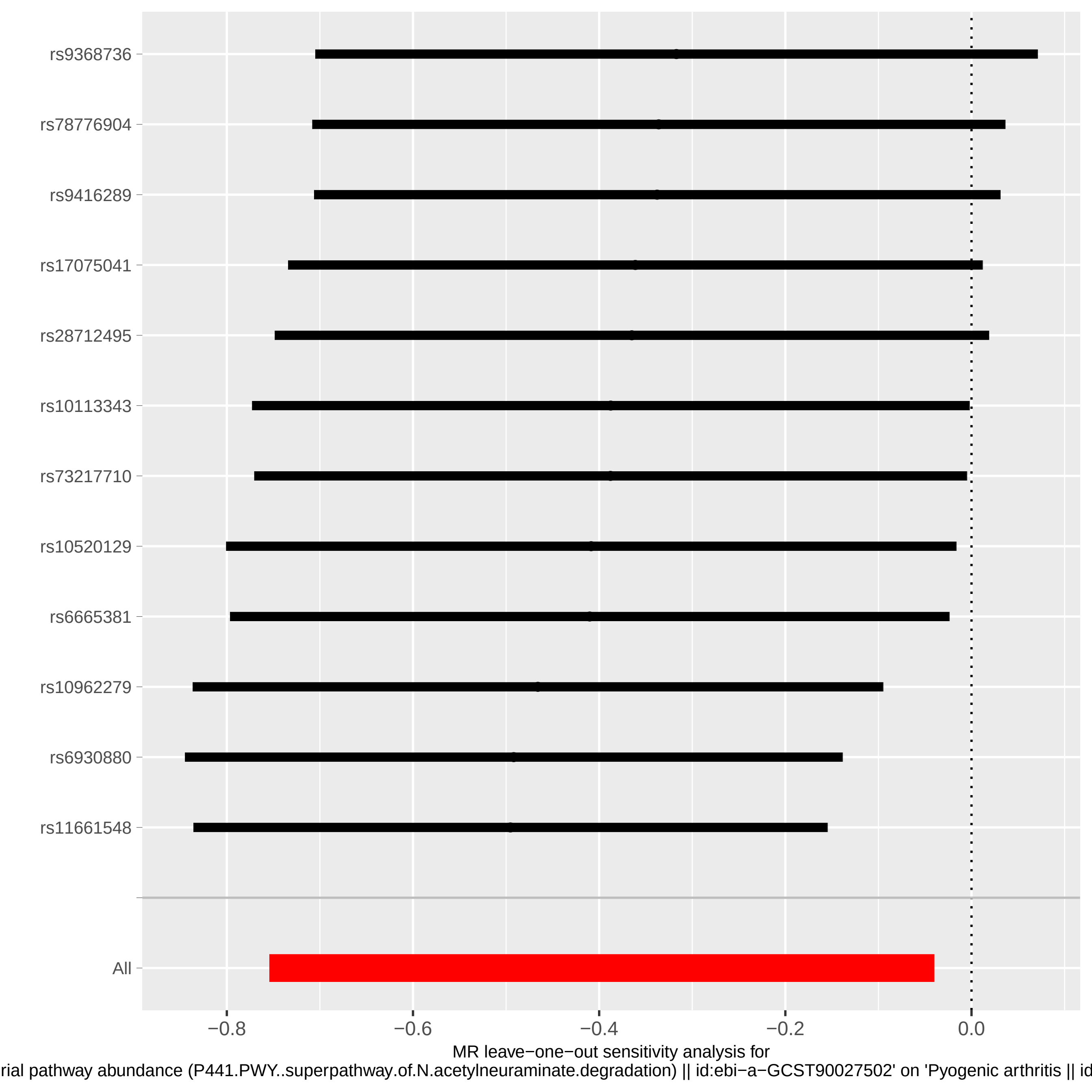
rs9368736
rs78776904
rs9416289
rs17075041
rs28712495
rs10113343
rs73217710
rs10520129
rs6665381
rs10962279
rs6930880
rs11661548
All
−0.8
−0.6
−0.4
−0.2
0.0
MR leave−one−out sensitivity analysis for
rial pathway abundance (P441.PWY..superpathway.of.N.acetylneuraminate.degradation) || id:ebi−a−GCST90027502' on 'Pyogenic arthritis || id
